# The Short-Term Retention of Depth

**DOI:** 10.3390/vision5040059

**Published:** 2021-12-08

**Authors:** Adam Reeves, Jiehui Qian

**Affiliations:** 1 Department of Psychology, Northeastern University, Boston, MA 02115, USA; 2 Department of Psychology, Sun Yat-Sen University, Guangzhou 510006, China; qianjh3@mail.sysu.edu.cn

**Keywords:** visual, visual working memory, distance

## Abstract

We review research on the visual working memory for information portrayed by items arranged in depth (i.e., distance to the observer) within peri-personal space. Most items lose their metric depths within half a second, even though their identities and spatial positions are retained. The paradoxical loss of depth information may arise because visual working memory retains the depth of a single object for the purpose of actions such as pointing or grasping which usually apply to only one thing at a time.

## 1. Depth and visual working memory

In nearly all past research on the visual storage of recently presented objects or symbols, the stimuli have been presented in the fronto-parallel or ‘picture’ plane. However, since the seminal paper by Xu and Nakayama (2007), [1], several researchers have investigated the role that depth information might play in short-term visual memory [2,3,4,5,6,7,8,9]. Here we review the contributions of visual depth to iconic memory and to visual working memory (VWM) as discovered so far. As an important preliminary, we first review evidence for the accurate perception of nearby spatial dimensions, especially depth, before discussing the retention of this information.

### Review Methodology

We searched the literature as follows. Xu and Nakayama [1] were the first to check whether WM capacity changed when items were spread over two depth planes compared to one depth plane. We therefore searched for the terms ‘iconic memory and depth’, ‘working memory and depth’, and, when those failed, ‘working memory’, in what we judged were the major journals in the field from 2007 to date. These include *Attention, Perception & Psychophysics;* *British Journal of Psychology; Journal of Experimental Psychology: General; Human Perception and Performance; Learning, Memory, and Cognition;* *Journal of the Optical Society of America A; Journal of Vision; Memory & Cognition; Proceedings of the National Academy of Sciences; Psychonomic Bulletin & Review; Psychological Review; Quarterly Journal of Experimental Psychology; Spatial Vision; Visual Cognition; and* *Vision Research.* The search terms were entered into the ‘search box’ for each journal, using the terminology of that journal (for example, using an acronym if necessary). References from before 2007, or from other journals, relied on our combined knowledge of the field, and were not searched systematically. In each paper returned by the search we determined the ‘depth effect’, namely, whether the authors reported that information in two or more depth planes was retained more accurately than information presented in one plane. Because we have been unable to find a sufficient number of papers outside our own laboratories, this review of the retention of depth in VWM may not be considered as ‘systematic’, as defined by PRISMA 2020. Since this review is necessarily slanted towards our own papers, we cannot claim to be unbiased, either in terms of the motivation for the research or its theoretical implications.

To summarize, we have found that the working memory for depth is remarkably, and perhaps surprisingly, poor, when compared to the working memory for other object properties. As far as we know, there are no published papers which contradict this generalization, which we attempt to explain in the Discussion.

In the papers that we review, the memoranda (the to-be-recalled visual stimuli) have all been presented inside peri-personal space (PPS), the space in our immediate surround in which we can act and manipulate objects without gross changes in body position. Object location (x, y, z) in PPS is represented well in perception, where x refers to the left–right position of an object and y to its up–down position, relative to gaze, while z is the distance from the head to the object. Beyond PPS, at long distances, the ground plane begins to dominate, and qualitative cues for depth, such as height in the field of view and interposition, start to replace the precision given by stereopsis and vergence. However within PPS, perception and action together give us access to all three dimensions. This fact implies that the memoranda are well situated perceptually and therefore available for retention rather than being poorly defined visually or otherwise noisy.

The experiments described here used stereoscopic disparity to generate depth, plus, sometimes, other depth cues. As stereopsis is remarkably precise, separations in depth corresponding to a few centimeters at arm’s length can be discriminated easily, and it therefore becomes possible to test for depth recall without being overly concerned with its encoding. Subjects typically sit before a flat display screen in the picture plane, with the display centered on the visual field. Gaze is held steady and eye movements are limited to micro-saccades. Nonius lines help ensure correct convergence. Absolute depth (z) in PPS, not just depth relative to the horopter or another object, is perceived accurately enough that subjects can reach out and touch an object portrayed in depth using disparity alone [10]. Within PPS, subjects with normal vision can encode location in the central visual field to within 2 min of arc in (x, y) and stereoscopic disparity (z) to within 2 min arc on the horopter and to within 4 min arc in near periphery, even off the horopter [11]. Such precision makes it easy for researchers to present memoranda in different locations or at different depths without too much concern for visibility.

In this review, the term ‘*spatial*’ refers to location (x, y). We assume that VWM stores the spatial parameters for each object. Thus individuals can point accurately at locations in space (x, y) that were briefly occupied by an object, well after it has been removed [12]. Here, the term ‘*depth*’ refers to z, the absolute or metric distance to the head, not to the thickness of an object, or to its distance from another object, or to its order in depth (closest to furthest away). The term VWMd refers to a possible visual working memory for the distances away (z) of various objects; it is questionable whether VWM and VWMd co-occur, overlap, or are distinct, as we discuss below. We only consider studies of the retention of *metric depth*, as there are no published studies of the retention of *depth order* as such.

## 2. Visual Short-Term Memory for (x, y)

In contrast to memory for depth, the short-term memory for visual stimuli presented in the picture plane (x, y) is well studied. In brief, there exists a visual short-term store (VSTS) which extends the ~500 ms duration of sensory (or ‘iconic’) memory [13] to more than 1 s [14,15,16,17,18], [19] section 2.3.4. For example, Lindsay-Wilson [20], in a study of iconic persistence, found that the identification of random-dot letter patterns whose halves were displaced in time survived initial iconic decay at well-above chance levels. Dual-task studies show that VWM and VSTS can function independently [21]. While the icon and VSTS enjoy relatively large capacities, only a few items enter VWM, either because of an attentional bottleneck (Boadbent, [22]) between VSTS and VWM, or because VWM has an intrinsically limited capacity [23,24]. The VWM capacity of four items reported by Luck and Vogel [24] varies somewhat with object complexity [6,25,26] and the disposition of visual attention [27,28,29]. Since studies of VWM typically employ retention intervals of 900 ms or more, exceeding the duration of iconic memory but incorporating items from VSTS, it is tempting to infer that any attentional bottleneck or capacity limitation starts to exerts its effects at some time between 0.5 and 0.9 s. 

It is clearly important to determine which visual memories survive an eye movement. Iconic memory does not [13], and VSTS (and therefore VWM) may lose all but an attended item following a saccade [23,30]. Indeed, any form of interruption of vision, even by a blank field, may suffice to erase visual memory [31,32]. No studies have assessed whether depth information survives a saccade or vergence movement, or some other interruption, so in this review, we can only review studies in which the eyes are held steady and the visual memory for items is not erased. Future studies may remedy this lacuna.

That memoranda are encoded with their spatial locations in iconic memory and in VSTS seems intuitive, and is presupposed when items are recalled by cues to spatial location [13,15] and many others. Spatial location may help to bind features in VMW [33,34], most likely at encoding [35]. Item color is bound to item location [36] (Gobell, Tseng, and Sperling, 2004), and item color and item shape only come apart in visual memory if attention is distracted [37,38]. Change detection is made more difficult if the spatial configuration of the probe display differs from that of the test display [39], as if spatial arrangement is held in VWM. True, it is possible to know that a particular object had been presented recently but not where, as in backward masking by pattern [19], so one cannot *a priori* exclude unlocated items from visual memory. However, the consensus that location (x, y) is stored along with each item in VWM makes it possible that depth (z) is also stored. We next consider whether this is the case or not, first in iconic memory, and then in working memory.

## 3. Depth Information in Iconic Memory

Reeves and Lei [9] were the first to study the contribution of depth to iconic memory, and concluded it was minimal. In their Experiment 1, rows of letters were either presented in one depth plane (‘flat’) or were separated into three depth planes. A partial report paradigm was used. Depth separation was induced using two depth cues, size (closer letters were made larger) and disparity. Depth had no effect on cued recalls at all, at any cue delay from –100 (pre-cue) to +700 ms (post-cue). In their Experiment 2, flat displays of four rows of letters were intermixed with concave displays in which the middle two rows appeared further away, or intermixed with both convex and concave displays, and again there was no depth effect. Their Experiment 3 encouraged a shift of attention between rows, and although items from rows 1 and 2 were slightly more likely to be transposed when the two rows were in different depth planes than in the same depth plane, no other transpositions showed any effect of depth. As none of the subjects had difficulty in seeing or reporting the depth manipulations, which were obvious, these authors concluded that iconic storage is effectively flat, consistent with the model of Sakitt [40]. It was not the case that partial reports could be increased by storing information from different depth planes in different locations in iconic memory. Either metric depth is not encoded in the icon, or it fades too rapidly to be useful, or it is retained but report is limited by a capacity limit or bottleneck.

Reeves and Lei [8] wondered whether the ‘flat’ icon they had found up to 700 ms was nevertheless sensitive to depth. They attempted to measure depth retention directly. Item information was made as simple as possible. Just four numerals, 1, 2, 3, and 4, were presented on each trial, in a well-spaced column running down the screen. Since only these four well-known numerals were presented, there could be no error in recalling their identities. Each numeral was presented in a different depth plane, but which numeral was in which depth plane was randomized. An arrow cue appeared next to fixation, either simultaneous with the numeral display, or just after it. Subjects reported the identity of the (single) numeral in the same depth plane as the arrow. They also counted backwards by threes during the retention interval to ensure that they did not rehearse verbally. 

In Sperling-type partial report experiments, subjects can report up to 11 letters from a spatial array of 12 letters [13]. In contrast, subjects in this experiment were very poor. For some, the stimulus duration had to be extended to 800 ms to retain any depth information, but as this permits vergence to change, data from these subjects were suspect. However eight subjects were able to do the task at better than chance with 200 ms displays. When the arrow cue was simultaneous with the numeral array, accuracy for the 200 ms subjects averaged 76% for recalling the depth of just one of four easy-to-see items. Accuracy dropped to 60%, and then increased slightly to 64% and 66% as the arrow cue was delayed from 0 ms to 200 ms, 700 ms, and 1700 ms (see Table 1, row 4). (Accuracy remained well above the chance level of 25%, so depth information was not absent from the icon, but it may have been too limited to aid the subjects of Reeves and Lei [9], who had reported from arrays of 9 or 12 items.) As the four items were distinct, well known, and perceived correctly on every trial, it was retaining their depths, not perceiving the depths or retaining them as objects, that was so difficult.

## 4. Depth Information in Visual Working Memory (VWM)

### 4.1. Partial Report

Reeves and Lei [8] had included longer delay periods along with the shorter delays appropriate for studying iconic memory. When the arrow cue was delayed by 1.7 s, overall accuracy averaged 66% rather the guessing rate of 25% expected from iconic decay, and critically was *better* than accuracy at 200 ms, by 2% for the four best subjects and 11% for the four worst ones. They modelled these data by assuming that the (albeit small) depth information in iconic memory decayed exponentially, but a slow integrative process also transferred some depth information into working memory, and that the subject recalled depth from the stronger of the two memory stores. To test this model, recall of depth was checked from 0 to 2s using the same arrow cue as before, but in addition, a four-item color memory load was applied before the trial and probed (by change detection) after the trial was over. This load was expected to have no effect on iconic memory but to strongly depress VWM [24]. However, to our surprise, the color load depressed depth recall in only one of six subjects, ruling out storage in VWM. These findings suggested a separate working memory for depth. The data for each subject were modeled by a fixed, rapid iconic decay plus a slow, individualized, transfer to a working memory for depth (call it VWMd). 

Whether VWMd exists as a separate memory from VWM, or refers to a distinct processing mode for items in VWM, was unclear, and anyway a single study is unlikely to be taken as definitive. However the authors noted that VWMd appeared to be unique, both in the lack of an effect of color load, and in that depth information accrues over time rather than decaying [8]. We return to this issue at the end of our review, simply using VWMd as a shorthand for ‘VWM for depth’ in the body of the review. 

### 4.2. Change Detection Task (CDT)

Xu and Nakayama [1] were the first to investigate whether VWMd capacity depended on the number of depth planes. They employed the change detection task (CDT) standard in studies of VWM. Their subjects detected a change in a random one of six colored squares presented for 200 ms and probed 1 s later. A verbal suppression task (rehearsing numerals) forced the subjects to employ visual memory during the retention interval. The subject reported whether the probe was of the same color and location as one of the test squares, or was different, changing to a new color in Experiment 1 and to a new location in Experiment 2A. In both cases, change detection improved by 4% when three items were placed on each of two depth planes, compared to when all six were on the same depth plane. Depth had no effect when subjects only had to recall the colors, not their locations. Grouping by motion and grouping within each depth plane also had no effect. They concluded that depth increased memory slightly for color-location bindings. Note however that for the effect of depth on accuracy, 4%, only occurred when the conditions were blocked, not randomized, so may this have merely reflected a change in strategy.

Qian and colleagues [5,41,42,43] tested whether VWM stores depth information using CDT. In [5], from one to six blue squares were presented for 800 ms, each in a different stereoscopically-defined depth plane, and probed 900 ms later. The test for recall was a probe square that could change in depth. The probe was either a single test square or a test square along with the other memory squares (the whole display). For the single square, the subjects were overall 71% correct in judging whether or not the probe was in the same depth plane as an original blue square, compared to 78% correct with the whole display. In both types of display, change detection became harder as the number of blue squares increased from one to six. This pattern of results is similar to that found with VWM, but the overall accuracy was lower, again suggesting that VWMd differs from VWM. Specifically, four observers also participated in a CDT task for color. The temporal parameters were the same. Mean accuracy was 97% for color and 73% for depth with a set size of four, and was 83% for color and 69% for depth with a set size of six. 

Wang et al. [42] employed the same CDT method as [5] but also varied fixation depth. All the items were equally visable at every depth. Change detection decreased away from the front plane when fixating the front, and decreased away from the back when fixating the back, but was worst at the middle plane when fixating the middle. Since attention is known to aid encoding into VWM [29], this result may be explained if attention is deployed to the front or back when either is fixated, but is split between the front and back when the middle is fixated. Unfortunately this is the only study of the potential role of attention in VWMd, an area needing further study. In particular, it is unknown whether attention aids retention or encoding or both in VWMd.

### 4.3. VWM Capacity in Depth

Capacity (K) can be estimated from Cowan’s [44] equation for single-probe displays, K = N(H−F), where N is the number of items and H and F are the hit and false alarm rates. For example, the 4% improvement due to splitting the N = 6 items over two depth planes found by Xu and Nakayama [1], if not due to a change in strategy, could reflect an increase in capacity. Given their hit and false-alarm rates, Cowan’s K = 3.7 when items were presented on two planes and 3.2 on one plane in their Experiment 1, and K = 2.5 for two planes and 2.0 for one plane in their Experiment 2A. Thus on average, K increased by half an item with depth, i.e., a gain of one item on half the trials or a gain of one item on all trials for half the subjects. The relatively small increase due to depth (4% or one half an item) may have been a ceiling effect in their Experiment 1, since WM capacity is limited to N = 4, but this was unlikely in Experiment 2A.

Qian et al. [3] found that separating the items in different stereoscopic depths hardly affected VWM after a 900 ms retention interval. They either presented all four or six items in one depth plane or split them evenly over two depth planes. Subjects judged whether the color of the probed item had changed after the retention interval or was the same, a CDT task. The estimated capacity (Cowan’s K) was 3.4 in both the one-plane and two-planes conditions, indicating that depth from disparity made no difference to capacity. However, when combining *two* depth cues, either disparity and relative size [3] or disparity and relative brightness-saturation [7], VWM capacity for six items improved by 0.4 items overall, from 3.5 in the one-plane condition to 3.9 in the two-planes condition. Capacity improved even more, by an estimated 0.7 items (assuming a common false alarm rate, F) for the closer depth plane, which was recalled better than the further depth plane. Critically, placing the two depth cues in conflict cancelled the memory benefit. The authors concluded that salient depth information can aid VWM capacity, by roughly one-half an item.

### 4.4. Set-Size Effect

Several authors have varied the set size, N, in studies of working memory. Accuracy generally declines with the number of items in VWM. However, Cowan’s capacity K= N(H−F) will be constant (at least over a limited range) if the decline in accuracy (H−F) is in proportion to N, providing some justification for assigning a specific capacity, K, to a memory store. 

Sarno et al. [45] compared one depth plane to two planes in a change detection task in which one of N items was displaced in depth. Across the 23 subjects in their Experiment 1, presenting items in two depth planes improved performance by 6% with a set size (N) of 5 items, but decreased performance by 8% with N = 3 items. Thus capacity (Cowan’s K) averaged 2.6 with both one and two depth planes, indicating no overall depth effect with N = 3 or 5. However, in their Experiment 2, the range of N was expanded to 8. Accuracy improved by 5% for N = 4 and N = 8, though not for N = 6, with capacity averaging 2.4 with two depth planes and 1.8 with one depth plane, for an improvement of 0.6 items. Importantly, in their Experiment 3, their 64 new subjects were segregated into 32 with higher scores (median accuracy 68%) and 32 with lower scores (median 62%). Both groups showed an increase in K for two depth planes compared to one depth plane when N = 4, but only the higher-scoring group also improved for N = 6 and 8. As there was no evidence for floor effects, the authors concluded that low VWM capacity individuals do not benefit from depth when memory is taxed. This is an important conclusion about individual differences in VWM; in contrast, Reeves and Lei [9] had found no effect of depth on iconic memory for either the best or worst half of their subjects. 

Chunharas et al. [2] found that presenting disks in separate depth planes slightly increased recall of their colors from VWM. The N = 2 target colors to be remembered were well separated in color space and appeared in the same or in different depth planes defined by disparity alone. In Experiment 1, target displays of 150 ms were followed by a probe (‘cue’) after 750 ms. Subjects adjusted a color wheel to match the color of the cued target. The standard deviation of the error in matching was 6% less when items were separated into two depth planes, indicating a benefit of depth. In their Experiment 2, CDT was employed. An array of N = 2, 4, 6, 8, or 12 disks were presented for 500 ms. One of the disks was probed for a color change after 900 ms. Accuracy (H−F) in Experiment 2 is given in Table 1, rows 5–8, by N and by number of depth planes. Accuracy decreased in proportion to N, so Cowan’s K was hardly affected by N and, critically, was entirely unaffected by depth for N = 2 to 8, averaging K = 2.15. However, K increased with depth when N = 12, from K = 1.6 to K = 1.8. The improvement when N = 12, though small, was slightly greater for the subjects with better stereoscopic acuity. 

The papers reviewed in this section make a case that both better stereoscopic acuity and an additional depth cue can enhance the effect of separating memory items in depth and thus increase the capacity of VWM, perhaps because metric depth is made more salient by better cues, or better depth cues slow decay. However these effects are small and only show up with high load. With only a few items (two or four), and only one depth cue (disparity), a null effect is commonly found. Recall, however, that these studies employing CDT only compared one depth plane with two. It is possible that separating items into more depth planes might demonstrate a greater benefit of depth on retention. 

### 4.5. Variations in Metric and Ordinal Depth

Qian et al. [4] presented an array of two or three blue squares for 800 ms, each in its own depth plane. Subjects reported whether or not the depth of one of them, a probe, had changed when tested 900 ms later. The magnitude of the depth change was varied, and either the probe retained its ordinal position among the memorized squares, or it switched its ordinal position with that of another memorized square. Order and metric distance interacted. With fixed δ, memory was better when the depth order was changed (mean hit rate = 0.89) than unchanged (mean hit rate = 0.67), but as increased, hit rate only increased (from 0.67 to 0.80) when depth order was fixed, not when it varied. This finding needs further study, but it suggests that depth relations, not just metric depths, are registered in VWMd. An exclusive emphasis on metric depth may be misleadingly limited.

### 4.6. VWM or VMWd?

Reeves and Lei [8] concluded from a partial report that depth was stored in a separate working memory (VWMd) which, unlike VWM, was not affected by an added color-memory load. Li et al. [43] repeated the CDT experiment of [5] with a variation designed to test whether VWM and VWMd really differed or not. During the retention interval they introduced a ‘retro-cue’ designed to bias visual memory towards one or other item. Souza and Oberauer [46] had suggested that a retro-cue not only strengthens an attended item in VWM but also helps remove unattended items, depending on the validity of the retro-cue. Li et al. [43] tested feature-based, spatial, and symbolic types of retro-cues. A memory array of four blue squares was shown for 800 ms, followed by a 200 ms blank ISI, a 300 ms retro-cue, and a further 800 ms delay until the probe. The subject had to report whether the depth plane of the probe matched, or did not match, the depth plane of the blue square in the memory array with the same spatial location. Accuracy in this task in Expt 1 was higher when the retro-cue was valid than when it was neutral or invalid, for all three types of retro-cue. Invalid cues were no worse than neutral ones, however, even though accuracy was in an appropriate range (76%) to show such an effect, which contrasts with results from studies showing invalidity effects with spatial arrays in VWM. The authors suggested that relational information is stored in VWMd but not in VWM, which could explain this difference.

## 5. Summary and Discussion

Summarizing some of the main findings (Table 1), Reeves and Lei [9] found no effect of separation in depth on retention up to 700 ms, and concluded that the icon (i.e., sensory memory) was flat. Xu and Nakayama [1] reported a weak (perhaps equivocal) positive depth effect on retention after 1 s. Chunharas et al. [2] probed VMW after 1 s and found that when load was high (N = 12), capacity increased by 0.2 items for colored items presented in two planes compared to one plane, but there was no depth effect when load was low. These three studies together indicate that short-term visual memory is either not affected by depth, or is affected slightly when load is high. It is not the case that placing items in two depth planes doubles capacity, as would be the case if memory were volumetric. At best, capacity is increased by one-half an item, not doubled; at worst, there is no improvement or even a slight loss. These memories are essentially flat.

That VWM memory for objects in the picture plane is considerably better than visual memory for depth is consistent with a similar comparison in visual search. Finlayson et al. [47] found that segregation in depth does not improve visual search for conjunctions and only improves feature searches when the target plane is known in advance and therefore can be selected. Godwin et al. [48] found that presenting opaque surfaces in one or two depth planes did not affect the accuracy of the visual search, although making the top surface transparent improved accuracy by up to 10%. Search times were not affected by depth in either condition. Thus depth information may support visual cognition as inferred from visual search as weakly as it appears to support visual memory

Nevertheless, information about depth is retained, even if overall capacity is hardly affected. For example, Reeves and Lei [8] found that depth recalls improved at 1.7 s instead of decaying exponentially with the icon, and suggested that some depth information (specifying one item or less) is transferred slowly to a specialized memory for depth (VWMd). Qian and Zhang [5] reported that the depth of a single item in one of six possible depth planes could be retained for 900 ms, although there were still numerous errors.

This summary poses two theoretical questions. The first is, why should depth effects on visual memory be so small, in every paradigm tested, given how critical depth is to immediate perception and to our memories of visual scenes? Clearly VMW does store location (x, y) and size (width, height, and length, w, h, l) of an object. The second question is less critical, but why should VWM differ from VWMd, that is, why should not VWM store z along with the other spatial parameters? Recall that adding a load of four colored items made no difference to the depth recalls reported by [8], even though a defining aspect of VWM is that all features (shape, color, etc.) sum to determine capacity [24], implying that VMWd may exist separately from VWM.

Possibly both questions can be answered if size and location parameters are coded ventrally, to individuate an object, whereas distance to the head (z) is coded dorsally [21,49,50] and, if used, will control action (grasping, pointing, and locomotion) rather than object identification. If so, VWM would be ventral and VMWd dorsal. We quote Ungerleider et al. [50]: “Within visual cortex, ventral stream areas are selectively involved in object vision, whereas dorsal stream areas are selectively involved in spatial vision. This domain specificity appears to extend forward into prefrontal cortex, with ventrolateral areas involved mainly in working memory for objects and dorsolateral areas involved mainly in working memory for spatial locations”. Necessarily, VMWd would have to retain all three coordinates (x, y, z), with a link to the object representation in VWM, to facilitate actions directed at an object, so location (x, y) would be represented in both memory systems. Note that the timings of VWM and VWMd may well differ. Whereas an immediate percept of location in external space (z, y, z) may control fast ballistic movements, as in rapidly pointing towards a target [12], a much slower transfer to VWMd may be required for correcting such movements over time. If so, the small capacity of VWMd would make sense, in that only one (or untypically, two) attended item is typically selected for action.

In the studies of VWMd undertaken so far, the definition of ‘item’ has been limited to discrete unrelated objects, but coherent groups of items can count as a single unit in VWM [51]. If this also applies to VWMd, a capacity of one ‘item’ may imply considerably more depth information in grouped objects, insofar as, we speculate, the group can be grasped or otherwise acted upon. Such experiments remain to be done.

For comparison with the memory for depth, Bradley and Pearson’s [52] subjects detected changes in the orientation, motion, or color of peripheral Gabor patches presented for 450 ms. A cue line indicated which of the 10 Gabors was the target. When the cue was delayed for a retention interval of 1 s to test the capacity of VWM, accuracy for noting a change was 55% for motion, 60% for orientation, and 65% for color. Critically, a demanding central task decreased retention at 1 s to chance, so they could conclude that retention of these attributes relied on attention and was therefore indicative of VWM [29]. Given these accuracy levels, WM capacity (Cowan’s K) for these attributes would be three items for color (as is commonly found) and two for orientation, but only one for motion, similar to our estimate of the capacity of VWMd. Motion is also encoded dorsally, in area MT. However, Öğmen, et al. [53] showed that a ‘bottleneck’ for motion processing preceded VWM, so this similarity may be accidental.

Such theorizing is rather premature, as the known facts offer so little constraint. Critically, although the positions, depths, and sizes of objects seen in PPS are initially encoded accurately by macular vision, sensory errors surely propagate over time. Studies of visual acuity in the 2-D picture plane suggest a two-stage process, a rapid decline followed by a much slower one. Thus Beard et al. [54], Figure 3, reported a three-fold decline in visual acuity when 25 ms test and reference stimuli were separated by an ISI of only 50 ms, compared to simultaneous presentation, although further increases in ISI to 500 ms had little effect. Fahle and Harris [55] reported a steady decline in spatial acuity of 1.8 times as the ISI was increased over a much longer period, from 1 s to 8 s, the time constant for this decay being about 14 s. Sheth and Shimojo [56] reported that distance to central vision (eccentricity) was compressed by about 25% after being held for 2 s in visual memory, a considerable distortion. Loomis et al. [57] asked subjects to walk towards a marker on the ground after shutting their eyes. The mean error in reaching the marker, as indicated by standard deviations plotted in their Figure 3, increased by 2.8 times as the length of the walk (and thus the retention interval) increased from 4 m to 10 m.

Unfortunately, few data exist with respect to comparisons of stimuli in different depth planes in PPS, and lacking this information makes it difficult to draw strong conclusions about VWMd. Even if the depths of the target and the probe are accurately encoded at presentation, they may drift apart in memory over 1 s or so [58], leading to a potential underestimate of the capacity of VWMd. Two studies of drift are directly relevant. Tanaka *et al*.’s [59] subjects saw a single target disk for 1 s projected 160, 200, or 240 cm away from the subject. They reported whether a probe disk presented 2 s later was closer or further away than the target. PSEs indicated that depth was compressed by about 83%, such that distant objects were remembered as closer, and close objects as more distant, than the actual depths. Zhang et al.’s [41] subjects adjusted the depth of a probe square to match that of a target square that had onset 900 ms earlier. The range of matched depths was contracted to about 90% of the actual depths as defined by disparity (and verified in perception). The contraction bias worsened to 80% as the initial array was increased to six items. It is therefore essential in studies of VWMd which involve items more than a few well-spaced depth planes that the subject’s recalls of the actual metric distances of the stimuli be tested, in order that the stimuli be spaced out sufficiently in depth for their depth planes to remain unambiguous. Reeves and Lei [8] cued one item in an array of four depth planes without testing for contraction over the 1 or 2 s interval from array to cue, which may have violated this constraint. We note that presenting objects in meaningful rather than random configurations may help reduce drift and increase the estimated capacity of VWMd, but there are no relevant studies to test this. Finally, although the literature has concentrated on metric depth, other depth parameters, such as depth order, may be better retained; again, such experiments need to be undertaken.

A final issue concerns the conception of working memory. Baddeley [60,61] built on an earlier conception of Broadbent [22] that the sensory buffer was followed by a bottleneck, or limited-capacity channel, such that only attended item could pass through it and receive further processing (e.g., recognition). This conception has become widespread as the distinction between the large capacity sensory store and the later restricted capacity has been confirmed in vision as well as hearing [62]. Baddeley [61] conceived of ‘further processing’ as an *executive* function, and gave examples of a phonological loop or a visuo-spatial sketchpad. However, no clear notion has arisen concerning any executive role concerning depth in VWMd. Even the role of attention on recall of depth remains to be fully characterized [43], and there are no studies of the effects of motor commands or other cognitive systems on VWMd. In an attractive theory due to Grossberg [63,64], items are selected by an attentional ‘shroud’ and then activated by resonances with long-term memory traces, but there are no empirical studies of how VWMd might be influenced in this manner. The conclusion of this review that VWMd is limited to the retrieval of the metric depth of just one item, appears to be well founded for segregated (ungrouped) items. Our suggestion that this is because of the need to facilitate actions like pointing or grasping that are applied to only one item at a time remains speculative.

## Figures and Tables

**Table 1:** Key findings.

Reference	Stimuli	Paradigm	Set Size	Display Time	Retention for	Accuracy
Xu & Naka-yama [1]	Squares	Change Detection, CDT	N = 6	200 ms	1000 ms	77% 1 or 2 planes (mixed) 81% 2 planes (blocked)
Reeves & Lei [9]	Letters	Partial report of 3 items (Expt. 1)	N = 9	50 ms	ISI = 0, 100, 300, 700 ms	0 100 300 700 ms (planes) 80%, 54%, 52%, 53% (one or two)
Chunharas et al. [2]	Colors	CDT (Expt. 2)	N = 2, 4, 6, 8, 12	500 ms	900 ms	N = 2, 4, 6, 8, 12 (planes) 80% 60% 40% 29% 15% (two) 80% 60% 39% 28% 13% (one)
Reeves & Lei [8]	Numeral	Partial report of 1 of 4 items	N = 4	best subjects; 200 ms	0, 200, 700, or 1700 ms delay	0 200 700 1700 ms 76%, 60%, 64%, 66%
Qian & Zhang [5]	Square	CDT	N = 1, 2, 4, 6	800 ms	900 ms	Single display 71%. Whole display 78%.
Qian et al. [4]	Square	CDT. δ = metric depth change	N = 2, 3	800 ms	900 ms	δ: small large 89% 93% (order changed) 67% 80% (unchanged)
Zhang et al. [41]	Square	Adjustment	N = 1, 6	800 ms	900 ms	90% contraction bias (N = 1) 80% contraction bias (N = 6)
Wang et al. [42]	Square	CDT	N = 2, 4	800 ms	900 ms	fixating middle plane, Front: 75%; Mid: 63%; Back: 73%
Li et al. [43]	Square	CDT; retro-cue	N = 4	800 ms	1300 ms	Cue valid 77%; Neutral: 71%; Invalid: 68%

δ = mtric depth change.
